# HMGA1 sensitizes esophageal squamous cell carcinoma to mTOR inhibitors through the ETS1-FKBP12 axis

**DOI:** 10.7150/ijbs.95595

**Published:** 2024-04-22

**Authors:** Jin-Rong Guo, Kai-Yue He, Jia-Li Yuan, Wang An, Wei-Tao Yin, Qiu-Tong Li, Li-Yuan Lu, Jing-Yu Yang, Meng-Jie Liu, Yu-Jia Li, Yuan Zhao, Qi Yang, Xin-Yuan Lei, Fan Gao, Lei Zhang, Dan-Hui Wu, Jun-Qi Li, Zi-Long Zhao, Huai Liu, Ling-Jun Zhu, Xiong-Yan Xiang, Qian-Hui Sun, Yong-Ping Jian, Zhi-Xiang Xu

**Affiliations:** School of Life Sciences, Henan University, Kaifeng, Henan Province, China.

**Keywords:** ESCC, HMGA1, FKBP12, ETS1, mTOR inhibitor, Rapamycin, resistance

## Abstract

Esophageal carcinoma is amongst the prevalent malignancies worldwide, characterized by unclear molecular classifications and varying clinical outcomes. The PI3K/AKT/mTOR signaling, one of the frequently perturbed dysregulated pathways in human malignancies, has instigated the development of various inhibitory agents targeting this pathway, but many ESCC patients exhibit intrinsic or adaptive resistance to these inhibitors. Here, we aim to explore the reasons for the insensitivity of ESCC patients to mTOR inhibitors. We assessed the sensitivity to rapamycin in various ESCC cell lines by determining their respective IC50 values and found that cells with a low level of HMGA1 were more tolerant to rapamycin. Subsequent experiments have supported this finding. Through a transcriptome sequencing, we identified a crucial downstream effector of HMGA1, FKBP12, and found that FKBP12 was necessary for HMGA1-induced cell sensitivity to rapamycin. HMGA1 interacted with ETS1, and facilitated the transcription of FKBP12. Finally, we validated this regulatory axis in *in vivo* experiments, where HMGA1 deficiency in transplanted tumors rendered them resistance to rapamycin. Therefore, we speculate that mTOR inhibitor therapy for individuals exhibiting a reduced level of HMGA1 or FKBP12 may not work. Conversely, individuals exhibiting an elevated level of HMGA1 or FKBP12 are more suitable candidates for mTOR inhibitor treatment.

## Introduction

Esophageal cancer (EC) is one of the most common malignant tumors globally. In 2020, its incidence ranked seventh among all malignancies, and its mortality ranked sixth. The global report for 2020 recorded over 600,000 new cases and more than 540,000 deaths from EC. The overall five-year survival rate is dishearteningly low, at only 20% 1,2.

Histologically, EC is mainly categorized into esophageal squamous cell carcinoma (ESCC) or esophageal adenocarcinoma (EAC) with the majority of recently diagnosed ECs worldwide attributed to squamous histological type. When the esophageal mucosa is exposed to carcinogens or experiences mechanical damage, epithelial cells undergo abnormal proliferation, ultimately leading to invasive cancer 3-5.

The molecular classifications of ESCC are unclear, contributing to its highly heterogeneous nature and variable clinical outcomes. Currently, there are no available prognostic biomarkers for this condition 6,7. Despite recent advancements in combination therapies, targeted treatment for ESCC remains limited, leading to an insufficient clinical management 8. Additionally, the precise molecular mechanisms underlying the etiology of ESCC are only partially understood, leading to the formidable challenge of treating EC. The most common treatment modalities for ESCC in clinical practice are chemotherapy and tyrosine kinase inhibitors. However, many ESCC patients exhibit intrinsic or adaptive resistance to these therapeutic agents. Therefore, it is imperative to identify the molecular mechanisms underlying drug resistance in patients or to develop new therapeutic targets.

The mTOR signaling pathway is frequently activated in ESCC, providing a theoretical basis for targeting mTOR in cancer treatment 9,10. Subsequent research efforts have resulted in the creation and synthesis of multiple small molecule inhibitors targeting this pathway. In addition, chemotherapy and tyrosine kinase inhibitor treatments may also induce an abnormal activation of the Akt-mTOR signaling pathway, leading to drug resistance 11,12. A common treatment strategy used in breast cancer and renal cell carcinoma involves the combination of chemotherapy drugs with mTOR inhibitors 13-18. However, some patients are insensitive to mTOR inhibitors, hindering the widespread application of this treatment strategy in ESCC patients. Elucidating the molecular mechanisms underlying the insensitivity of ESCCs to mTOR inhibitors would help us better identify patients who are sensitive to mTOR inhibitor treatment, thereby enabling more personalized therapy.

The mTOR inhibitor rapamycin was the first small molecule to be translated from the laboratory to the clinic for targeting the mTOR pathway 10,19. As of now, three generations of mTOR inhibitors have been developed. High-dose rapamycin is being used in clinical trials for cancers with aberrant activation of mTOR and its signaling pathways 20-23. Rapamycin exerts its effects by binding to the FKBP12 protein, forming a complex that interacts with the FKBP-rapamycin-binding (FRB) region of mTOR 24, preventing other substrates from entering the active site and inhibiting mTORC1 activity 25. This inhibition by rapamycin on mTORC1 pathway leads to suppressed protein synthesis and inhibited cell growth, both of which contribute to tumor regression 26.

The major pathways leading to mTOR resistance reported to date include: 1, different ATP-binding cassette (ABC) transporter proteins, which function as drug efflux pumps, such as ABCA1 and ABCB1 27,28. 2, mTOR inhibitors primarily suppress cap-dependent translation rather than cap-independent translation 29,30. Mutations or loss of 4EBP lead to the resistance of mTOR inhibitors 31,32. 3, Researchers have identified over 30 mutations of mTOR associated with diverse forms of human cancer 33,34. Among them, mutations of C1483F, E1799K, and S2215Y lead tumors more sensitive to mTOR inhibitors, whereas mutations of S2035F, F2018L, and A2034V are associated with pharmacological resistance 35,36. 4, Several other oncogenic pathways regulate the sensitivity of mTOR inhibitors. PTEN / PIK3A mutations usually make tumors sensitive to mTOR inhibitors, while the opposite is true for BRAF/ KRAS mutations 37-41.

HMG proteins, a family known for its high abundance of chromatin-binding factor 42-44, were initially identified in the 1970s within calf thymus tissue through salt extraction and solubility testing in trichloroacetic acid 45. HMG proteins, such as HMGB, HMGN, and HMGA, constitute a group of molecules characterized by their basic nature and low molecular weight. These proteins exhibit swift migration when subjected to polyacrylamide gel electrophoresis, hence earning the label "high-mobility group proteins”.

The HMGA proteins differ from other HMG families by possessing three AT-hook domains, facilitating their attachment to the minor grooves within B-form DNA with high affinity 42-44,46,47. HMGA proteins also contain an acidic carboxyl-terminal tail rich in serine and proline residues, which facilitates protein-protein interactions 44. HMGA1 competes with histone H1 for DNA binding, leading to the structural changes in the minor groove of DNA, assembly of transcription factors and enhancers, and hence regulation of gene expression 48-57.

Studies have shown that HMGA1 is involved in chemoresistance to platinum-based drugs (cisplatin), imatinib, 5-fluorouracil (5-FU), methotrexate, gemcitabine, and cyclophosphamide in various cancers 59-68. However, the relationship between HMGA1 and responses to mTOR inhibitors in cancer has not been reported. In this study, we unexpectedly uncovered a connection between HMGA1 and rapamycin resistance in ESCC, elucidated the molecular mechanisms underlying HMGA1's modulation of mTOR inhibitor sensitivity, with the identification of the pivotal factor FKBP12 through the transcriptome analysis. Further investigation revealed that HMGA1, through its interaction with ETS1, enhanced the binding of the latter to the promoter region of FKBP12, thereby facilitating FKBP12 transcription. Consequently, this process ultimately heightens the responsiveness of ESCC to rapamycin treatment.

## Materials and Methods

### Human samples

Human ESCC tumor and peritumoral specimens were collected from The People's Hospital of Anyang City. Usages of human samples were in accordance with the principium delineated in the Declaration of Helsinki and approved by the Henan University Review Board.

### Animals and treatments

C57BL/6 mice were obtained from SIPEIFU Biotech Limited (Beijing, China). Approval for conducting the animal experiments was obtained from the Ethics Committee at the Institute for Advanced Biomedical Studies in Henan University. The animals were treated in a humane manner following the guidelines delineated in the Guide for the Care and Use of Laboratory Animals published by the NIH. Mice were accommodated in a climate-controlled setting (maintained at 23°C ± 2°C) with a 12 h light / 12 h dark cycle. For the experiments detailed in this report, mice aged 7-9 weeks were utilized.

### Cell culture

ESCC cell lines, KYSE30, KYSE510, KYSE70, KYSE140, TE13, EC109, and EC9706, were purchased from ATCC and grown under 5% CO2 and 21% O2 in RPMI 1640 (PM150110, Pricella) including 10% FBS (S711-001s, Lonsera). Verification of the ESCC cell lines was conducted based on cellular morphology, and authentication was performed using STR DNA analysis. Additionally, tests were performed to confirm that there was no mycoplasma contamination in cells.

### Lentiviral transduction

The constructed lenti-V3 plasmid as well as the two auxiliary plasmids, pMD2.G/psPAX2, were transfected into 293 cells with lip2000 (11668019, Invitrogen) under the second generation of lentiviral packaging system. Subsequently, the supernatant of 293 cells was collected and subjected to centrifugation at 2,000 rpm for 10 minutes and passed through a 0.45 μm filter (SLHV033RB, Millipore) to remove impurities. When target cells reached 80% fusion, the packaged viruses were mixed with polybrene (6 μg/ml) and dropped into the cell culture medium. Cells were selected with puromycin. Confirmation for the expression of target gene in the cells was achieved through RT-PCR and Western blot analysis.

### Cell transfections

DNAs were transfected into cells with Lipofectamine (Thermo, 11668-019) according to the manufacturer's instructions. For siRNA transfections, INTERFERin (Polyplus, product code: 101000028) was utilized following the instructions from the manufacturer.

### Western blot

Western blots were executed following the procedures outlined previously 69. In brief, cells were lysed in NP40/RIPA buffer at a concentration of 1 × 10^4 cells per μl. Proteins were separated using SDS-PAGE gel and transferred into PVDF membranes (Millipore, IPVH00010). Membranes were exposed to the first and second antibody diluted in 1% BSA and detected with an ECL kit (Solarbio, PE0010).

### Antibodies and chemicals

Antibodies against HMGA1 (ab129153), HMGA1-ChIP Grade (ab252930), and FKBP12 (ab2918) were purchased from Abcam. Antibodies against HMGA1 (sc-393213) and FKBP12 (sc-133067) and mouse IgG (sc-2025) were purchased from Santa Cruz Biotechnology. Antibodies against S6 ribosomal protein (#64108) and phospho-S6 ribosomal protein (#81736) and rabbit IgG (#2729), mTOR (#2983), phospho-mTOR (#5536), eIF4EBP1(#9452), and phospho-eIF4EBP1(#9456) were purchased from Cell Signaling Technology (Danvers, MA). Antibody against beta actin (81115-1-RR) was purchased from Protein Tech (Wuhan, China). Rapamycin was obtained from MedChemExpress (HY-10219).

### Immunofluorescence

Cell cultures were established on 35mm confocal dishes 24 h prior to the experiment. Following this, the cells were fixed in 4% paraformaldehyde for 15 min, permeabilized with 0.3% Tween-100 for 17 min, and washed with PBS for 3 times with 5 min each. After blocking with 1% BSA, cells were incubated with primary antibodies for 2 h, followed by PBS washing for 3 times with 5 min each. Fluorescent secondary antibodies (Abcam, ab150077, ab150115) were then applied into the cells for the detection. ProLong Diamond mounting medium containing DAPI (Thermo Fisher Scientific, 62248) was used for sealing the slide and staining DNA. Imaging was conducted using a confocal laser scanning microscope (Zeiss). Image analysis was carried out using ZEN (Zeiss) and ImageJ (NIH).

### Colony formation assay

Cells were planted in the 6-well plate at a low density (900 cells per well) and maintained for 11 days before being fixed with 4% formaldehyde in PBS. Following fixation, cells were stained with 0.1% crystal violet and washed with PBS. Colonies with 50 or more cells were counted.

### Cell viability assay and IC50 calculation

Cells were planted into wells in a 96-well plate (1,000 cells/well) and cultured for 24 h. Different concentrations of mTOR inhibitor or control PBS were added to the plate. Cells were incubated for another 48 h in the incubator. Afterward, CCK-8 was added to each well and the plate was placed in the incubator for 1.5 h before measured using a TECAN with an absorbance of 450 nm. The results obtained from 3 experiments were imported into Graphpad 10. Concentrations were log-transformed, and IC50 values were calculated using nonlinear regression analysis.

### RT-qPCR

RNAs of ESCC cells were extracted with TRIzol reagent (Merck, T9424) and reverse-transcribed into cDNA by RT SuperMix (Vazyme, R333-01). qPCR was performed on Roche 480. Reactions were run in RT SYBR Fluor qPCR (Qiagen, 330513). The cDNA synthesis was performed within a total capacity of 20 microliters under typical cycling parameters. Relative genetic expression underwent normalization utilizing GAPDH as a reference gene and computed via the Comparative CT Method (2^ΔΔCT).

### ChIP-PCR analysis

ChIP-PCR was conducted following the methods described previously 69. Briefly, 1% paraformaldehyde was added to the cells for cross-linking for 10 min, followed by termination of the cross-linking using glycine. After washing of the cells with PBS, SDS lysis solution was added to the cells and the lysate was sonicated for 7 min utilizing a Diagenode bioruptor device. Subsequently, the chromatin extract was immunoprecipitated using an HMGA1 antibody (Abcam, ab252930). Enriched DNA was subsequently isolated using the DNA Purification Kit (QIAGEN, 69504) after the ChIP complex was de-crosslinked at 65°C for 6 hours. Finally, changes in DNA levels were detected by aforementioned PCR.

### Immunohistochemistry

Tissue specimens were sectioned to a thickness of 4 micrometers. Slides were immersed in 1X citrate-based unmasking solution and subjected to microwave heating until boiling commenced. Subsequently, slides were maintained at a temperature just below boiling (between 94°C and 97°C) for 15 minutes. Following this, slides were allowed to cool off on the benchtop for 40 min before staining using the UltraSensitive™ SP (Mouse/Rabbit) IHC Kit (Maxim, China). The antibodies utilized for immunohistochemistry were as follows: anti-HMGA1 (diluted 1:500, sc-393213, Santa Cruz), anti-phospho-S6 ribosomal (diluted 1:1200, #81736, Cell Signaling Technology), and anti-FKBP12 (diluted 1:1200, ab2918, Abcam).

### Co-immunoprecipitation

Proteins were extracted from human ESCC cells using NP40 lysis buffer supplemented with PhosSTOP phosphatase inhibitor (Roche) and a protease inhibitor blend (Roche). The quantification of extracted proteins was performed using the BCA assay (Solarbio, China). For co-immunoprecipitation (co-IP), 800 μg of lysates were incubated overnight at 4°C with 5 μg of antibody or IgG, followed by the addition of 26 μl of Protein A/G-Agarose Beads (#9863, CST) and rotation for 1.5 hours. The beads underwent five wash cycles in immunoprecipitation buffer, followed by mixing with 15 μl of 1× loading buffer for western blot analysis.

### Luciferase assay

The promoter regions of *FKBP1A* were amplified through PCR and subsequently integrated into the pGL3 basic vector to form reporter constructs. Various segments of the FKBP12 promoter or modified vectors were generated through subsequent cloning by PCR. KYSE30 and 293T cells were seeded into 96-well plates and transfected with pGL3 constructs. After 48 hours, the luciferase activity of the *FKBP1A* promoter was measured using the Duo-Lite Luciferase Assay System Kit (DD1205-01, Vazyme).

### RNA sequencing

Total RNAs from ESCC were extracted using Trizol. The quality of RNA isolated from ESCC cells was assessed using the Bioanalyzer 2100 system. All samples with an RNA Integrity Number (RIN) greater than 7 were utilized for library construction and subsequently sequenced on the Illumina Novaseq platform by Frasergen (Wuhan, China).

### Statistics

All data presented in the figure represent the results obtained from three or more biological replicates. The t-test was employed for comparing the means of two groups, while one-way ANOVA was utilized for comparing means among multiple groups of samples. (* p < 0.05; ** p < 0.01; *** p < 0.001).

## Results

### Expression of HMGA1 is correlated with the sensitivity of ESCC to rapamycin

**Figure 1:**
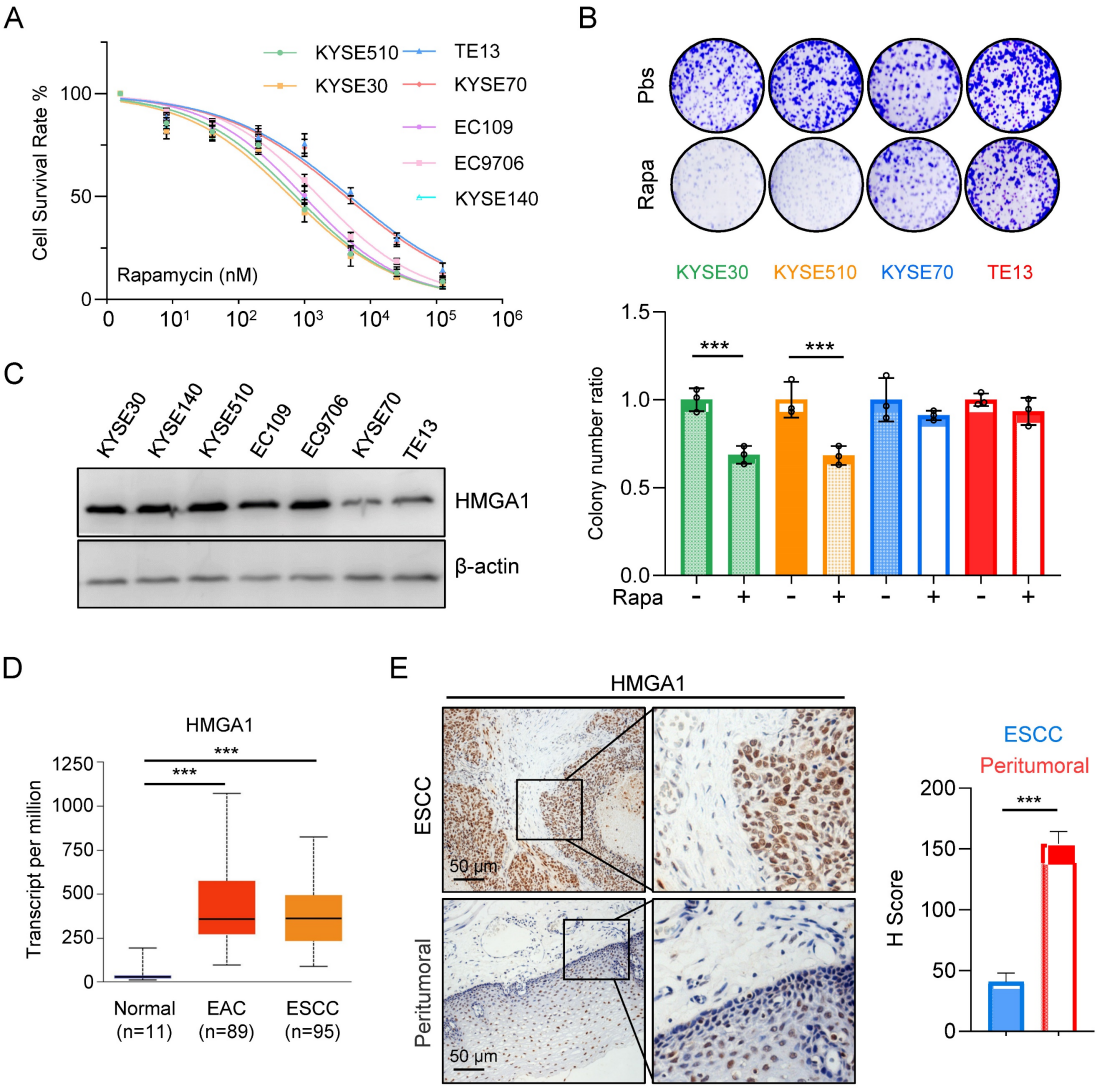
** Expression of HMGA1 is correlated with the sensitivity of ESCC to rapamycin. (A)** Treatment of different ESCC cells with rapamycin for 48 hours. Cell viability was assessed using CCK8 experiments. Results represent average of 3 experiments with 3 replicates. IC_50_ (KYSE510: 745.5 nM; KYSE30: 621.4 nM; TE13: 4900 nM; KYSE70: 4242 nM; EC109: 983.5 nM; EC9706: 1671 nM; KYSE140: 831.8 nM). **(B)** Colony formation assay was performed on different ESCC cells after 36 hours of treatment with 1 μM rapamycin. **(C)** Expressions of HMGA1 were analyzed in different esophageal cancer cell lines using western blot. β-actin was detected as an internal control.** (D)** HMGA1 in normal esophagus, esophageal adenocarcinoma (EAC), and ESCC in a dataset from the UALCAN database. **(E)** Representative images of HMGA1 immunohistochemical (IHC) staining in tumor tissues and adjacent tissues of esophageal cancer patients, scale = 50 μm.

Aberrant activation of mTORC1 has been consistently observed across various cancer types, including ESCC. While mTORC1 inhibitors have demonstrated efficacy in clinical settings against a spectrum of tumors, their sustained use is often hindered by the rapid development of resistance among patients, with the underlying mechanisms of resistance remaining largely elusive. In our investigation of ESCC's sensitivity to mTOR inhibitors, we initially assessed the half-maximal inhibitory concentration (IC50) of rapamycin across different esophageal cancer cell lines. Our findings revealed a notably elevated IC50 for KYSE70 and TE13 cells compared to other cell lines (Fig. 1A).

Subsequently, we exposed various ESCC cell lines to rapamycin and conducted a colony formation assay. The results illustrated a marked reduction in survival colonies following rapamycin treatment in KYSE30 and KYSE510 cells (Fig. 1B). Conversely, the reduction in survival colonies was only marginal for KYSE70 and TE13 cells (Fig. 1B), suggesting a heightened resistance to rapamycin in these cell lines relative to several other ESCC cell lines.

The observed phenomenon prompted a recollection of our prior experimental findings (Fig. 1C), wherein the expression levels of HMGA1 in various ESCC cell lines strikingly correlated with their susceptibility to rapamycin. Notably, cells exhibiting elevated HMGA1 expression demonstrated heightened sensitivity to rapamycin, whereas those with diminished HMGA1 levels displayed resistance to rapamycin treatment (Fig. 1A-C).

For the assessment of HMGA1 expression in EC, we employed the UALCAN database for our analysis. The results unveiled a markedly elevated level of HMGA1 in both ESCCs and EACs in comparison to normal esophageal tissue (Fig. 1D). To further corroborate these findings, we conducted an immunohistochemical staining (IHC) on tumor tissues obtained from ESCC patients. Our analysis revealed a conspicuous overexpression of HMGA1 in tumor tissues of ESCC patients (Fig. 1E). These findings strongly suggest that HMGA1 is prominently expressed in ESCC, a phenomenon potentially linked to heightened sensitivity to rapamycin.

### Aberrant expression of HMGA1 in ESCC cells leads to altered responsiveness to rapamycin

**Figure 2:**
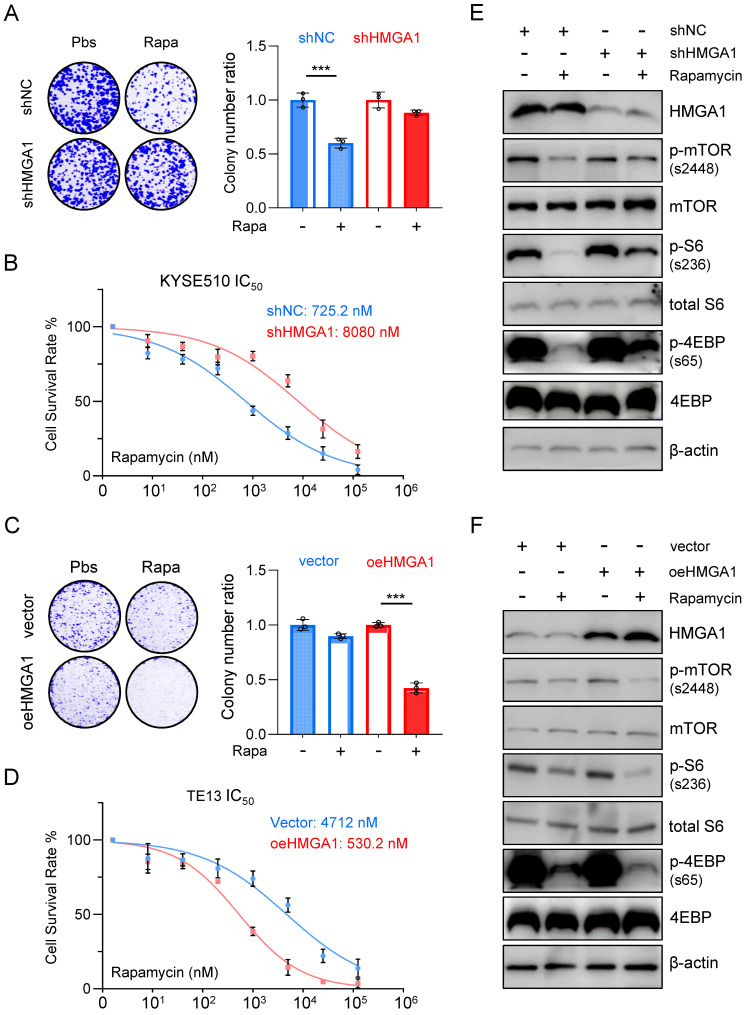
** Aberrant expression of HMGA1 in ESCC cells leads to altered responsiveness to rapamycin. (A)** Colony-forming units in KYSE510 cells with or without HMGA1 silencing (control vs shHMGA1). Cells were simultaneously treated with 1 μM rapamycin for 48 h. Representative images of colony formation are shown. **(B)** KYSE510 cells with or without HMGA1 knockdown were treated with rapamycin in a concentration gradient (starting at 8 nM and increasing fivefold each time). Cell viability was measured using the CCK8 assay. **(C)** Colony-forming units in TE13 cells with or without HMGA1 overexpression (vector vs oeHMGA1). Simultaneously, the cells were treated with 1 μM rapamycin for 48 h. Representative images of colony formation are shown.** (D)** TE13 cells with or without HMGA1 overexpression were treated with rapamycin in a concentration gradient (starting at 8 nM and increasing fivefold each time). Cell viability was measured using the CCK8 assay. **(E)** HMGA1, FKBP12, mTOR downstream effectors, and beta-actin (loading control) in KYSE510 cells transduced with shHMGA1 were detected with immunoblot. Extracts of ESCC cells were collected 24 h after 1 μM rapamycin treatment. **(F)** HMGA1, FKBP12, mTOR downstream effectors, and beta-actin (loading control) in TE13 cells transduced with lentivirus-expressed HMGA1 were detected with immunoblot. Extracts of ESCC cells were collected 24 h after 1 μM rapamycin treatment.

To further elucidate the impact of HMGA1 expression on the susceptibility of ESCC to rapamycin, we utilized shRNA to knock down HMGA1 expression in KYSE510 cells and subjected them to rapamycin treatment. Remarkably, the depletion of HMGA1 in KYSE510 cells resulted in a reduced susceptibility to rapamycin. Specifically, under equivalent rapamycin concentrations, shHMGA1 cells exhibited greater resistance to treatment in both colony formation and cell viability assays compared to shRNA control cells treated with rapamycin (Fig. 2A). Notably, we observed that HMGA1 depletion in KYSE510 cells led to a notable increase in the IC50 concentration (the concentration at which 50% of cells cease proliferating upon rapamycin treatment) compared to KYSE510 cells without HMGA1 knockdown (8,080 nM vs. 725.2 nM; P < 0.001) (Fig. 2B).

Conversely, overexpression of HMGA1 using lenti-V3 in TE13 cells heightened the susceptibility of cells to rapamycin treatment. Specifically, at identical rapamycin concentrations, oeHMGA1 cells exhibited a reduced survival in colony formation and a lower cell viability compared to empty vector-transduced cells (Fig. 2C). Additionally, we observed that HMGA1 overexpression in TE13 cells led to a decreased IC50 concentration (530.2 nM vs. 4,712 nM; P < 0.001) (Fig. 2D).

Furthermore, we investigated whether the differences in IC50 values resulting from altered HMGA1 expression were specific to rapamycin or applicable to other mTOR inhibitors. To complement our findings, we selected the third-generation mTOR inhibitor, rapalink-1, for additional validation. Results revealed that in KYSE510 cells, HMGA1 knockdown led to an increase in the IC50 value for rapalink-1 (30.52 nM vs. 296.8 nM; P < 0.001), while conversely, overexpression of HMGA1 in TE13 cells resulted in a decrease in the IC50 value for rapalink-1 (255.7 nM vs. 18.73 nM; P < 0.001). In summary, our observations indicate that increased HMGA1 expression renders cells a sensitivity to both aforementioned mTOR inhibitors, whereas decreased expression of HMGA1 leads to heightened tolerance of ESCC cells to mTOR inhibitors.

To further confirm the finding that HMGA1 sensitizes ESCC cells to rapamycin, we examined the phosphorylation levels of downstream effectors of mTOR across different cellular lines. In KYSE510 cells, rapamycin treatment notably decreased the phosphorylation of mTOR (S2448), S6 (S236), and 4EBP (S65). However, upon depletion of HMGA1, the inhibitory effect of rapamycin on the phosphorylation of mTOR downstream effectors was attenuated (Fig. 2E). In TE13 cells, characterized by relatively weak endogenous expression of HMGA1, rapamycin treatment significantly reduced the level of mTOR-S2448, S6-S236, and 4EBP-S65. Interestingly, overexpression of HMGA1 augmented the inhibitory effect of rapamycin on the phosphorylation of mTOR downstream effectors (Fig. 2F).

### HMGA1 upregulates *FKBP1A* (FKBP12)

**Figure 3:**
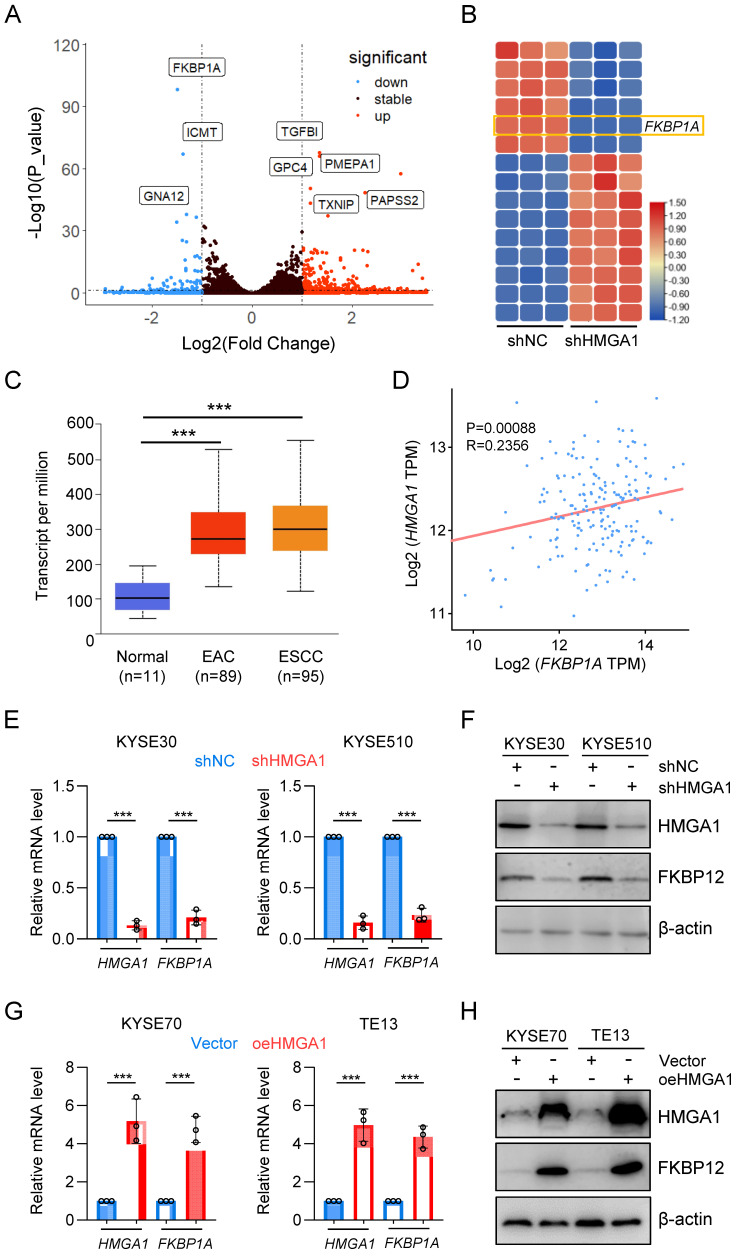
**HMGA1 upregulates *FKBP1A* (FKBP12). (A)** RNA-seq identifies deregulated genes in KYSE30 cells with HMGA1 silencing. A volcano plot is presented. **(B)** Heatmap shows top 15 significantly DEGs in the RNA-seq using HMGA1 silencing and control KYSE30 cells. **(C)** *FKBP1A* in normal esophagus, esophageal adenocarcinoma, and ESCC in a dataset from the UALCAN database. **(D)** A positive association was observed between the expression of *FKBP1A* and HMGA1 in esophageal cancers from the GEPIA database. **(E)** Relative FKBP12 expression from 3 independent experiments conducted in triplicate qPCR assay in KYSE30 and KYSE510 cells, with or without HMGA1 silencing (shcontrol vs shHMGA1). GAPDH was used as a control for calculating the relative mRNA. **(F)** Western blots for the detection of HMGA1, FKBP12, and beta-actin (loading control) in KYSE30 and KYSE510 cells with or without HMGA1 silencing (shcontrol vs shHMGA1). **(G)** Relative FKBP12 expression from 3 independent experiments conducted in triplicate qPCR assay in KYSE70 and TE13 cells, with or without HMGA1 overexpression (control vs oeHMGA1). GAPDH was used as a control for calculating the relative mRNA. **(H)** Western blots for the detection of HMGA1, FKBP12, and beta-actin (loading control) in KYSE70 and TE13 cells with or without HMGA1 overexpression (control vs oeHMGA1).

To elucidate the impact of HMGA1 on the susceptibility of ESCC cells to rapamycin, we established stable HMGA1-silenced KYSE30 and KYSE510 cell lines and conducted RNA sequencing on these cells. Unsupervised hierarchical clustering revealed distinct separation between HMGA1-expressing (control) and HMGA1-silenced cells (Fig. S1A). Differentially expressed genes (DEGs) identified from RNA-seq analysis (FDR < 0.05, log2 [fold change] > 1) comprised 202 upregulated genes and 57 downregulated genes. For enhanced visualization of differential gene expression, we adjusted the y-axis of the volcano plot (*HMGA1* was excluded from the plot due to its low p-value) (Fig. 3A). The heatmap highlighted the top 15 markedly DEGs (Fig. 3B). Among them, *FKBP1A* drew our attention (FPKM value, Fig. S1B). FKBP12, encoded by *FKBP1A*, serves as an intracellular receptor for rapamycin and is likely a pivotal factor in HMGA1's modulation of cell sensitivity to rapamycin. Analysis of the UALCAN database indicated a higher expression of *FKBP1A* in both ESCC and EAC, consistent with the *HMGA1* expression pattern (Fig. 3C). To validate the correlation between *HMGA1* and *FKBP1A*, we examined the GEPIA database and identified a positive correlation between *HMGA1* and *FKBP1A* in ESCC (p < 0.00056) (Fig. 3D). The HMGA1-dependent expression of FKBP12 was confirmed at both mRNA and protein levels in KYSE30 and KYSE510 cells with HMGA1 knockdown (Fig. 3E, F). In contrast, overexpression of HMGA1 in KYSE70 and TE13 cells, which have relatively low endogenous HMGA1 levels, led to an upregulation of FKBP12 (Fig. 3G, H). Collectively, our findings support the notion that HMGA1 upregulates *FKBP1A* (FKBP12).

### Silencing FKBP12 phenocopies HMGA1 deficiency, and restoring FKBP12 partially reverses the impact of HMGA1 depletion

**Figure 4:**
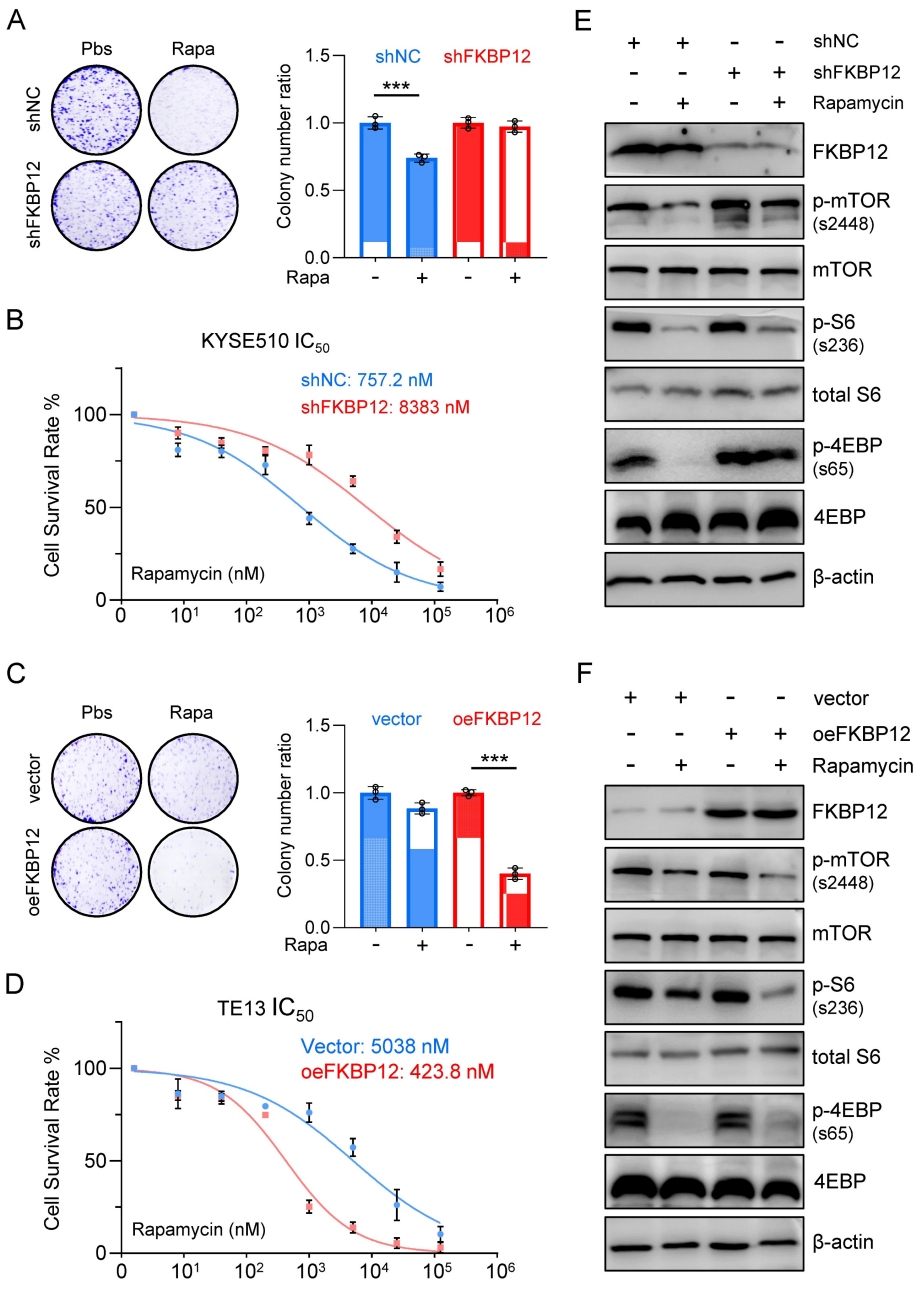
** Silencing FKBP12 phenocopies HMGA1 deficiency. (A)** Colony-forming units in KYSE510 cells with or without FKBP12 silencing (control vs shFKBP12). Simultaneously, the cells were treated with 1 μM rapamycin for 48 h. Representative images of colony formation are shown. **(B)** KYSE510 cells with or without FKBP12 knockdown were treated with rapamycin in a concentration gradient (starting at 8 nM and increasing fivefold each time). Cell viability was measured using the CCK8 assay. **(C)** Colony-forming units in TE13 cells with or without FKBP12 overexpression (vector vs oeFKBP12). Simultaneously, the cells were treated with 1 μM rapamycin for 48 h. Representative images of colony formation are shown. **(D)** TE13 cells with or without FKBP12 overexpression were treated with rapamycin in a concentration gradient (starting at 8 nM and increasing fivefold each time). Cell viability was measured using the CCK8 assay. **(E)** FKBP12, mTOR downstream effectors, and beta-actin (loading control) in KYSE510 cells transduced with shFKBP12 were detected with immunoblot. Extracts of ESCC cells were collected 24 h after 1 μM rapamycin treatment. **(F)** FKBP12, mTOR downstream effectors, and beta-actin (loading control) in TE13 cells transduced with lentivirus-expressed FKBP12 were detected with immunoblot. Extracts of ESCC cells were collected 24 h after 1 μM rapamycin treatment.

To assess the necessity of FKBP12 for HMGA1 function, we employed shRNAs to deplete FKBP12 in KYSE510 cells. Our findings revealed that FKBP12 silencing resulted in a reduced susceptibility of cells to rapamycin, as evidenced by a lesser decrease in clone-forming ability and cell viability (Fig. 4A, B). Moreover, the concentration of rapamycin required to achieve 50% inhibition of proliferation in KYSE510 cells increased upon FKBP12 depletion (757.2 nM vs. 8,383 nM; P < 0.001) (Fig. 4B).

In contrast, overexpression of FKBP12 increased the sensitivity of TE13 cells to rapamycin, as demonstrated by a greater decrease in clone-forming ability and cell viability (Fig. 4C, D). Furthermore, the concentration of rapamycin required to achieve 50% inhibition of proliferation in TE13 cells decreased upon FKBP12 overexpression (5,038 nM vs. 423.8 nM; P < 0.001) (Fig. 4D). Consistent with our findings regarding HMGA1 manipulations, in KYSE510 cells with FKBP12 silencing, rapamycin treatment exhibited weaker inhibition on the downstream effectors of mTOR compared to control cells (Fig. 4E). In contrast, in TE13 cells with FKBP12 overexpression, rapamycin treatment demonstrated a stronger inhibition on the downstream effectors of mTOR compared to control cells (Fig. 4F). Taken together, our results suggest that FKBP12 plays a pivotal role in determining the susceptibility of these ESCC cells to rapamycin.

**Figure 5:**
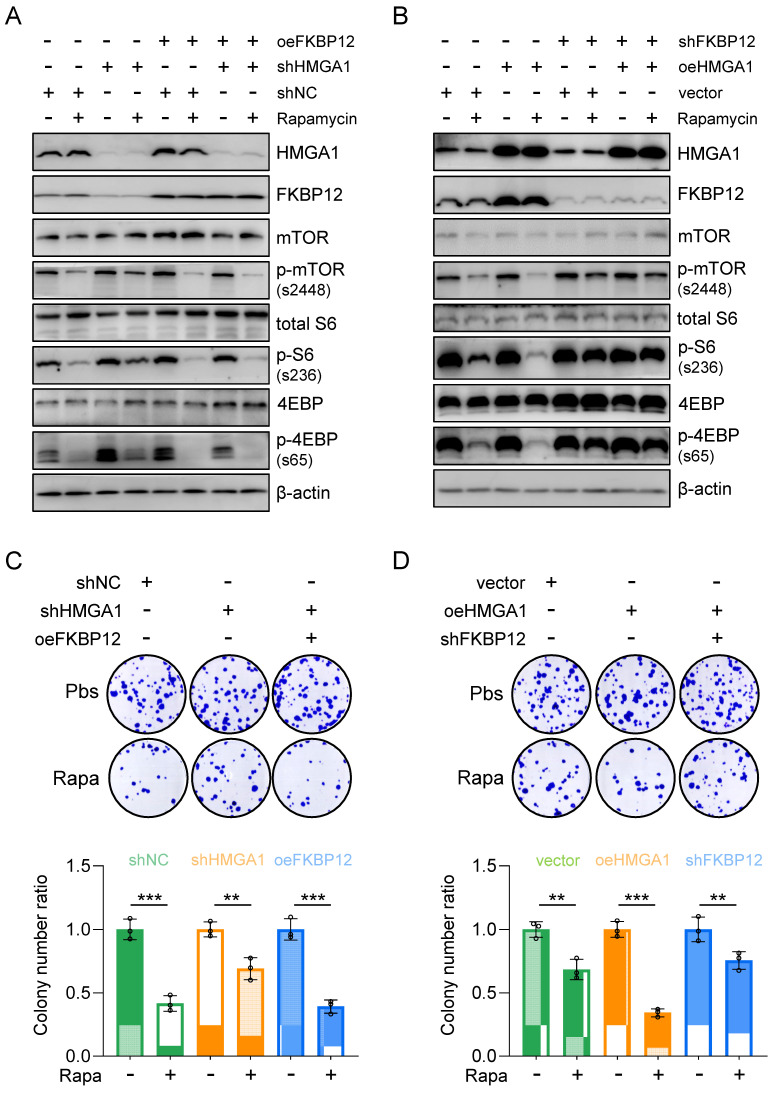
** Restoring FKBP12 partially reverses the effects of HMGA1 depletion on cell sensitivity to rapamycin. (A)** KYSE510 shRNA ctrl and shRNA-HMGA1 (control vs shHMGA1) cells were transduced with pcDNA3.1/FKBP12 and treated with 1 μM rapamycin. Whole cell extracts were isolated for the detection of HMGA1, FKBP12, mTOR downstream effectors, and beta-actin (loading control) with western blot. **(B)** TE13 empty vector and HMGA1 overexpression (vector vs oeHMGA1) cells were transduced with FKBP12-shRNA and treated with 1 μM rapamycin. Whole cell extracts were isolated for the detection of HMGA1, FKBP12, mTOR downstream effectors, and beta-actin (loading control) with western blot. **(C)** KYSE510 shRNA ctrl and shRNA-HMGA1 (control vs shHMGA1) cells were transduced with pcDNA3.1/FKBP12 and treated with 1 μM rapamycin. Colony formation assay was performed in the cells. Representative images of colony formation are shown in the upper panel. Colonies with 50 or more cells were counted. Calculations of relative colonies in each treatment (combination of treatments) are shown in the lower panel. **(D)** TE13 empty vector and HMGA1 overexpression (vector vs oeHMGA1) cells were transduced with FKBP12-shRNA and treated with 1 μM rapamycin. Colony formation assay was performed in the cells. Representative images of colony formation are shown in the upper panel. Colonies with 50 or more cells were counted. Calculations of relative colonies in each treatment (combination of treatments) are shown in the lower panel.

To assess whether reinstating FKBP12 expression could reverse the cell susceptibility to rapamycin resulting from HMGA1 silencing, we generated KYSE510 cells with HMGA1 suppression while simultaneously inducing FKBP12 overexpression to re-establish FKBP12 expression levels comparable to those in parental cells (Fig. 5A). Subsequently, we gauged the cell susceptibility to rapamycin by assessing the activation status of downstream effectors of mTOR (Fig. 5A). Our findings revealed that following HMGA1 silencing, the rapamycin-mediated reduction in mTOR-S2448, S6-S236, and 4EBP-S65 was attenuated. However, enforced FKBP12 expression reinstated rapamycin-induced suppression of mTOR-S2448, S6-S236, and 4EBP-S65 in HMGA1-depleted cells (Fig. 5A).

Furthermore, shRNA-mediated silencing of FKBP12 was employed in HMGA1-overexpressed TE13 cells to observe the cellular response to rapamycin. Our investigations unveiled that HMGA1 overexpression initially heightened the efficacy of rapamycin in inhibiting mTOR downstream effectors. However, upon FKBP12 depletion, HMGA1 failed to exert this effect (Fig. 5B). These findings suggest that the role of HMGA1 in modulating cell sensitivity to rapamycin is contingent upon FKBP12. Notably, FKBP12 partially rescues the phenotypes associated with HMGA1 depletion, consequently augmenting cell susceptibility to rapamycin.

To further validate the role of FKBP12 in HMGA1-sensitized cellular response to rapamycin, we assessed the clonogenic potential of cells treated with rapamycin under the manipulation of HMGA1 and FKBP12. In KYSE510 cells, although HMGA1 silencing alleviated rapamycin-reduced cell clonogenicity, enforced expression of FKBP12 restored rapamycin's capacity to inhibit cell clonogenicity (Fig. 5C). Similarly, in TE13 cells, overexpression of HMGA1 was unable to sensitize cells to rapamycin in the presence of shRNA FKBP12 (Fig. 5D). Collectively, our data suggest that FKBP12 is required for the effect of HMGA1 on rapamycin sensitivity.

### HMGA1 facilitates the transcription of *FKBP1A* by interacting with ETS1 and aiding ETS1 in binding to the promoter of *FKBP1A*

**Figure 6:**
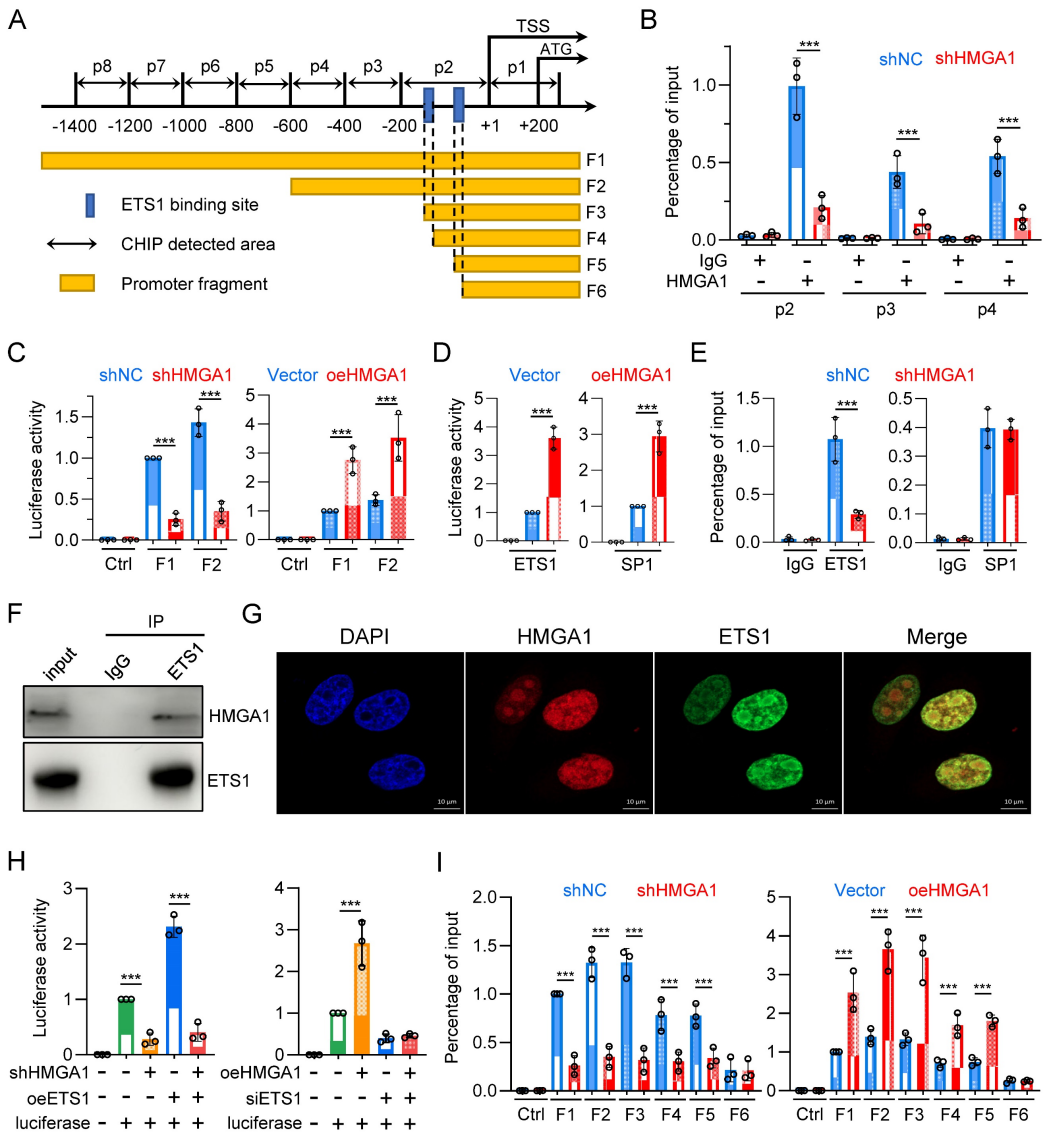
** HMGA1 facilitates the transcription of *FKBP1A* by interacting with ETS1 and aiding ETS1 in binding to the promoter of *FKBP1A*. (A)** Schematic representation of human *FKBP1A* region, with the transcription start site (TSS) and translation start site (ATG) indicated in the figure. p1-p8 represent the ChIP detection regions, and F1-F6 are the promoter fragments cloned into pGL3-basic vector. The blue box marks the computationally predicted ETS1 binding region within 200 base pairs upstream of the TSS. **(B)** ChIP PCR was performed to detect the binding of HMGA1 to the *FKBP1A* promoter region in KYSE30 cells with or without HMGA1 silencing. HMGA1-chip antibody and non-specific control IgG were used in the ChIP assay to assess HMGA1 binding to the target region. Results are expressed as the percentage recovered from the total input DNA (% input), and the experiments were performed in triplicate across three independent trials. **(C)** Dual-luciferase reporter assay was conducted to evaluate the functionality of *FKBP1A* promoter in KYSE30 cells with or without HMGA1 silencing and the promoter activity of *FKBP1A* in 293T cells with control and HMGA1 overexpression using promoter fragments F1/F2. **(D)** Dual-luciferase reporter assay was conducted to evaluate the functionality of *FKBP1A* promoter in 293T cells with control and overexpression of SP1 and ETS1, using promoter fragments F1. **(E)** ChIP PCR was performed to detect the binding of ETS1/SP1 to the *FKBP1A* promoter region in KYSE30 cells with or without HMGA1 silencing. ETS1/SP1-chip antibody and non-specific control IgG were used in the ChIP assay to assess ETS1/SP1 binding to the target region. Results are expressed as the percentage recovered from the total input DNA (% input), and the experiments were performed in triplicate across three independent trials. **(F)** Co-immunoprecipitation (Co-IP) was performed to validate the interaction between HMGA1 and ETS1. Whole cell lysates of KYSE30 cells were used, and immunoprecipitation was carried out using anti-ETS1 antibody and mouse IgG as a control. The presence of HMGA1 was detected using anti-HMGA1 antibody. **(G)** Immunofluorescence was employed to investigate the subcellular localization of HMGA1 and ETS1 in KYSE30 cells. Scale bar = 10 μm. **(H)** Dual-luciferase reporter assay was conducted to evaluate the functionality of *FKBP1A* promoter in KYSE30 shRNA ctrl and shRNA-HMGA1 (control vs shHMGA1) cells. Cells were transduced with pcDNA3.1/ETS1 and promoter fragments F1/F2 were used for the determination of luciferase activity (left panel). Dual-luciferase reporter assay was also conducted to evaluate the functionality of promoter *FKBP1A* in 293T empty vector and HMGA1 overexpression (vector vs oeHMGA1) cells. Cells were transduced with FKBP12-shRNA and promoter fragments F1/F2 were used for the determination of luciferase activity (right panel). **(I)** Dual-luciferase reporter assay was conducted to evaluate the functionality of *FKBP1A* promoter in KYSE30 shRNA ctrl and shRNA-HMGA1 (control vs shHMGA1) cells, using promoter fragments F1-F6 (left panel). Dual-luciferase reporter assay was also conducted to evaluate the functionality of *FKBP1A* promoter in 293T cells with control and overexpression of HMGA1 (vector vs oeHMGA1), using promoter fragments F1-F6 (right panel). The promoter fragments include the following. F1, F2. main binding regions of HMGA1 in the *FKBP1A* promoter; F3. Fragment containing two ETS1 binding sites; F4. Fragment with one ETS1 binding site truncated; F5. Fragment with the sequence between two ETS1 binding sites truncated; F6. Fragment with both ETS1 binding sites truncated. Data were obtained in triplicate in 3 independent experiments.

To determine whether HMGA1 directly activates *FKBP1A*, we designed 8 pairs of primers (corresponding to p1 - p8) targeting the -1400/+200 region of the *FKBP1A* promoter and tested whether HMGA1 binds to these areas (Fig. 6A). The results showed that HMGA1 bound to regions p2, p3, and p4 (-600/+1), with the highest binding affinity in the p2 region in the ChIP assay (Fig. 6B). Moreover, when we silenced HMGA1, its binding to the *FKBP1A* promoter obviously decreased (Fig. 6B), (full results in Fig. S3A). To functionally validate that the residency of HMGA1 in the *FKBP1A* promoter region directly regulates the promoter activity of *FKBP1A*, we evaluated the activity of the dual-luciferase reporter gene, and found that the activity of the constructed plasmid (F1: -1902/+621) decreased after silencing HMGA1 (Fig. 6C, left panel). Based on the results of the ChIP experiment, we also constructed a luciferase reporter vector with a promoter fragment (F2: -600/+56), which showed a decreased activity after silencing HMGA1 (Fig. 6C, left panel). Furthermore, we overexpressed HMGA1 in HEK293T cells using pcDNA3.1 vector. In contrast to the results from HMGA1 silencing, overexpression of HMGA1 noticeable increased the luciferase activity of the *FKBP1A* promoter fragments (including F1 and F2) in HEK293T cells (Fig. 6C, right panel).

As a structural transcription factor, HMGA1 cannot independently change the promoter activity of target genes. To determine how HMGA1 promotes the transcription of *FKBP1A*, we used ALGGEN and Jaspar databases to analyze the potential transcription factors with binding motifs contained in the promoter fragment F1 and found that the frequency of enrichment of SP1 and ETS1 was high. To verify whether SP1 and ETS1 regulate the promoter activity of *FKBP1A*, we constructed plasmids overexpressing SP1 and ETS1, respectively, and transfected them into HEK293T cells. Both obviously enhanced the luciferase activity of the *FKBP1A* promoter fragment F1 (Fig. 6D). This indicates that both SP1 and ETS1 regulate the promoter activity of *FKBP1A*.

To clarify whether HMGA1 regulates the promoter activity of *FKBP1A* through SP1 or ETS1 or both, we tested whether SP1 and ETS1 bind to the promoter regions of p1-p8. The results showed that SP1 bound to the p3 region, and ETS1 bound to the p2 region. However, only the binding of ETS1, but not SP1, to the promoter region of *FKBP1A* obviously decreased after silencing HMGA1 in the ChIP assay (Fig. 6E) (full results in Fig. S2B, C). To elucidate how HMGA1 facilitates the binding of ETS1 to the *FKBP1A* promoter region, we examined the interaction between HMGA1 and ETS1 proteins. We confirmed the interaction between HMGA1 and ETS1 in KYSE30 cells through co-immunoprecipitation assay (Fig. 6F). Additionally, in cell immunofluorescence staining, we observed the subcellular localization of HMGA1 and ETS1 in KYSE30 cells, indicating that both HMGA1 and ETS1 were predominantly localized in the cell nucleus and exhibited a significant co-localization (Fig. 6G). These results indicate that HMGA1 promotes the transcription of *FKBP1A* by interacting with ETS1 and assisting ETS1 in binding to the *FKBP1A* promoter.

To determine whether HMGA1-mediated transcriptional activation of *FKBP1A* is dependent on ETS1, we conducted a rescue experiment. We transfected an expression plasmid of ETS1 into KYSE30 with HMGA1 silenced. The results suggest that the luciferase activity of *FKBP1A* promoter fragment F1 was reduced following HMGA1 silencing (Fig. 6H). Overexpressing ETS1 increased the luciferase activity of *FKBP1A* promoter by 2.3 times. However, after silencing HMGA1, overexpressing ETS1 only increased the luciferase activity of the *FKBP1A* promoter fragment F1 by 1.45 times (Fig. 6H, left panel). The results indicate that high expression of HMGA1 makes ETS1 more likely to bind to the *FKBP1A* promoter, rather than changing the expression level of ETS1, enhancing the transcription of *FKBP1A*.

In addition, we overexpressed HMGA1 while using siRNA to reduce the expression of ETS1 in HEK293T cells. The results indicated that overexpression of HMGA1 obviously reinforced the luciferase activity of *FKBP1A* promoter fragment F1. However, when ETS1 was silenced by siRNA, HMGA1 could no longer increase the luciferase activity of the *FKBP1A* promoter fragment F1 (Fig. 6H, right panel). These results demonstrate that ETS1, to a large extent, mediates the function of HMGA1 in promoting the transcription of *FKBP1A*.

To identify the specific binding sites of ETS1 in the *FKBP1A* promoter region, we performed a motif analysis on the *FKBP1A* promoter region -200/+1 based on ChIP experimental results (Fig. 6I) using databases in ALGGEN and Jaspar. We selected two binding sites with the highest scores, both with the sequence GCTTCCGG, located at -173/-165 and -147/-139, respectively, and named them EBS1 and EBS2 (ETS1 binding sites 1/2). We then constructed a series of truncated mutants (Fig. 6A): *FKBP1A* promoter fragment F2 containing the HMGA1 binding site, *FKBP1A* promoter fragment F3 containing only EBS1 and EBS2, *FKBP1A* promoter fragment F4 missing only EBS1 compared to F3, *FKBP1A* promoter fragment F5 missing the sequence between EBS1 and EBS2 compared to F4, and *FKBP1A* promoter fragment F6 missing only EBS2 compared to F5 (Fig. 6A). The results showed that the knockdown of HMGA1 obviously decrease the luciferase intensity of *FKBP1A* promoter fragments F1/2/3/4/5, but not F6 (Fig. 6I). The degree of fluorescence signal attenuation in fragments F4/5 after HMGA1 silencing was lower than that in fragments F1/2/3 (Fig. 6I, left panel). We also evaluated the dual-luciferase reporter in HEK293T cells, and found that overexpression of exogenous HMGA1 obviously increased the fluorescence intensity of *FKBP1A* promoter fragments F1/2/3/4/5, but the increase in fragments 4/5 was less than that in fragments 1/2/3, and fragment 6 could not even be increased (Fig. 6I, right panel). These results suggest that the presence of both EBSs (fragments F1/2/3) results in the highest level of HMAG1 regulation of *FKBP1A* promoter activity, and that the degree of regulation decreases as the number of EBSs decreases (fragments 4/5). When EBS was lost (fragment 6), HMGA1 also lost its regulation on *FKBP1A* promoter. Overall, the expression of ETS1 and the presence of its binding site are essential for HMGA1 to regulate *FKBP1A* transcriptional activity.

### Inhibiting HMGA1 leads to reduced sensitivity of ESCC to rapamycin *in vivo*

**Figure 7:**
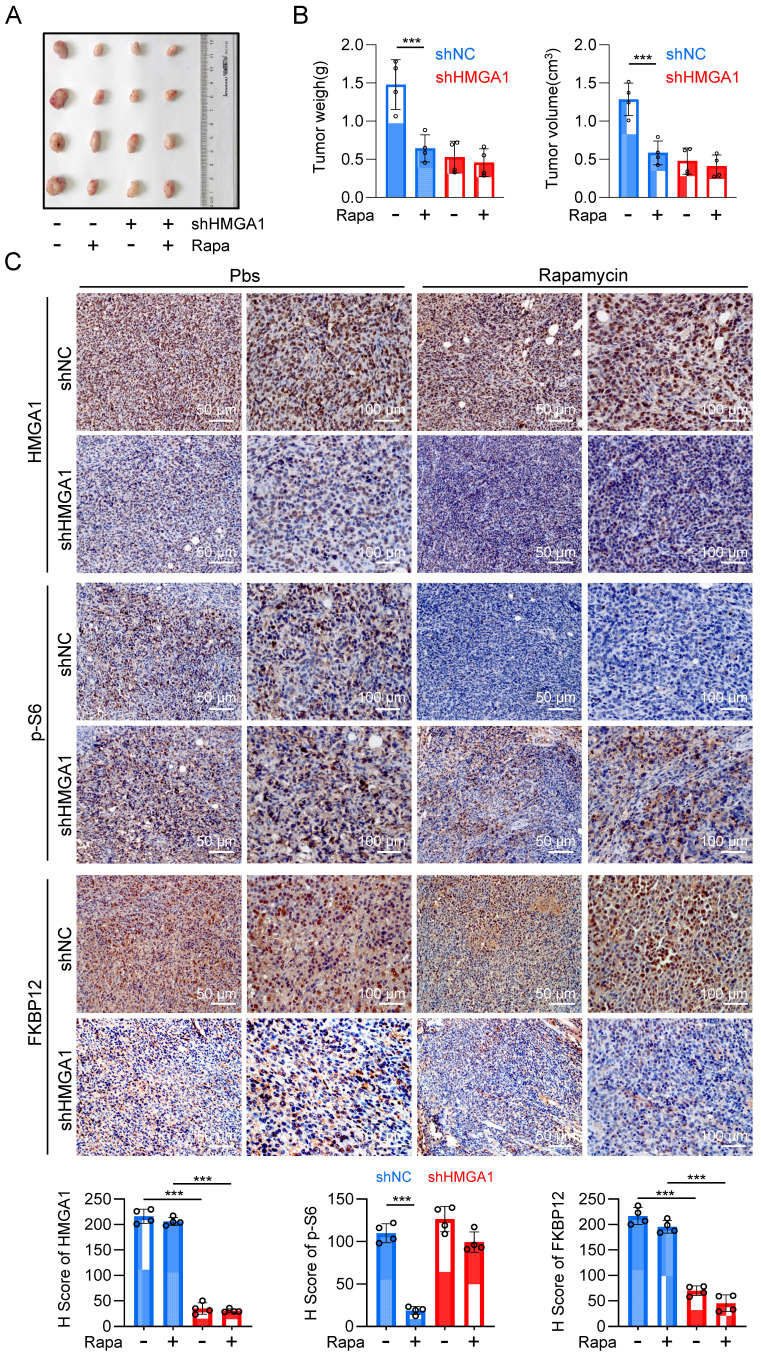
** Inhibiting HMGA1 leads to reduced sensitivity of ESCC to rapamycin in vivo. (A)** A syngeneic subcutaneous ESCC model in mice. AKR mouse esophageal cancer cells with HMGA1 silenced or unsilenced were injected subcutaneously into the axilla of C57BL/6 mice, followed by intraperitoneal injection of rapamycin (2 mg/kg) after one week, with an additional injection every 48 hours. **(B)** Subcutaneous tumor weight and volume in syngeneic mice at endpoint were calculated for each group of mice (n = 4) using the formula (length × width²) × 0.52 and presented as mean ± SEM. **(C)** IHC staining of HMGA1, p-S6, and FKBP12 in the syngeneic tumor tissues (slice = 4 μm). Representative IHC staining images were presented, scale = 50 μm (left) and 100 μm (right).

With the purpose of validating the *in vivo* significance of HMGA1-induced upregulation of FKBP12 and resultant susceptibility of cells to rapamycin, we established a syngeneic tumor transplantation model by inoculating mouse-derived esophageal cancer cell AKR, along with AKR cells stably silenced for HMGA1 using shRNA, into the axillary region of C57BL/6 mice. Rapamycin was administered for the treatment when the tumor volume was greater than 100 mm^3^. Silencing HMGA1 obviously decreased the proliferation of the syngeneic tumor, resulting in smaller tumor size and mass compared to the control group (Fig. 7A, B). Furthermore, we observed that rapamycin therapy effectively inhibited the growth of tumors in the control group, but not in HMGA1-knocked-down tumors (Fig. 7A, B). These findings indicate that the silencing of HMGA1 confers a resistance to rapamycin in esophageal cancer syngeneic tumors.

Immunohistochemical staining of tumor sections revealed knockdown of HMGA1 resulted in the decrease of HMGA1 and FKBP12 in murine tumors (Fig. 7C). To evaluate the sensitivity of tumors to rapamycin treatment, we also detected the phosphorylation of mTOR downstream effector, S6. In the control group, rapamycin treatment markedly reduced the phosphorylation of S6 (S236). However, after HMGA1 knockdown, when the intracellular expression of FKBP12 was low, rapamycin treatment no longer reduced the phosphorylation level of S6 (Fig. 7C). Interestingly, the silencing of HMGA1 in the syngeneic tumors exhibited a greater inhibition of esophageal cancer growth (Fig. 7A, B) compared to HMGA1 silencing in cells cultured (Fig. 2A), implying that HMGA1 acts as a more potent oncogene (tumor promoter) in the tumor microenvironment *in vivo*. In conclusion, our study highlights that HMGA1 regulates the sensitivity of ESCC to rapamycin through the modulation of FKBP12.

## Discussion

EC exhibits a persistently high mortality. Effective treatment options for EC remain scarce. Concurrently, esophageal tumors demonstrate a significant heterogeneity among different patients, leading to considerable variations in clinical treatment outcomes. Personalized precision therapy is of paramount importance in reducing EC mortality and enhancing the efficacy of therapeutic agents. However, our current understanding of molecular alterations in ESCC is incomplete and has not been fully translated into clinical practice 70-76. Therefore, deepening our knowledge of precise molecular events driving ESCC heterogeneity and their correlation with clinical information will foster the discovery of novel treatment targets and assist us to identify patients who might be of drug resistance to certain therapies.

Previous research has primarily focused on whether and how the mTOR signaling pathway is abnormally activated in tumors, such as ESCC. It was found that this pathway is upregulated to fulfill the demands of the tumor for rapid proliferation-related protein synthesis. Consequently, numerous mTOR inhibitors have been developed and employed clinically for cancer treatment. However, there exist substantial variations in the treatment efficacy, with certain patients displaying resistance to mTOR inhibitor therapy. Therefore, we aim to investigate the underlying reasons for patient resistance to mTOR inhibitors.

Initially, we assessed the sensitivities of different ESCC cell lines to the mTOR inhibitor rapamycin. To our astonishment, we observed a seemingly correlated relationship between the expression levels of HMGA1 in cells and their responsiveness to rapamycin. Specifically, cells with relatively higher HMGA1 expression exhibited a low IC_50_ of around 1 μM, whereas cells with lower HMGA1 expression displayed an IC_50_ of over 4 μM (Fig. 1A). To validate the finding that HMGA1 modulated the susceptibility of ESCC cells to rapamycin, we conducted experiments involving the silencing or overexpression of HMGA1 and unequivocally demonstrated that the susceptibility of ESCC cells to rapamycin does indeed fluctuate in correspondence with alterations in the expression of HMGA1 (Fig. 2). We then conducted an RNA-seq analysis and identified that FKBP12, encoded by *FKBP1A* gene, mediated HMGA1-enhanced susceptibility of ESCC cells to rapamycin. We further characterized a transcriptional regulatory mechanism by which HMGA1 modulates FKBP12 expression. HMGA1 interacted with ETS1 and facilitated ETS1-mediated transcription of FKBP12.

The identification of *FKBP1A* and its association with HMGA1 sheds light on the underlying molecular mechanisms that govern the susceptibility of ESCC cells to rapamycin. Further studies exploring the intricate interplay between HMGA1 and *FKBP1A* could potentially lead to novel therapeutic strategies targeting this signaling pathway to improve the effectiveness of rapamycin-based treatments in ESCC and possibly other cancer types as well. Indeed, we validated the HMGA1-ETS1-FKBP12 axis in the susceptibility of ESCC tumors to rapamycin *in vivo* and demonstrated that HMGA1 deficiency rendered the transplanted tumors insensitive to rapamycin. Therefore, we speculate that mTOR inhibitor therapy for individuals exhibiting reduced HMGA1 or FKBP12 levels may not work. Conversely, individuals exhibiting elevated levels of HMGA1 or FKBP12 in their tumors are more suitable candidates for mTOR inhibitor treatment.

HMGA1 is frequently overexpressed in aggressive and advanced-stage malignant tumors. Jang developed a prognostic prediction model using sncRNA to classify different EC patients into high/low-risk groups, analyzing suitable targeted drugs for them 77. Their findings indicated that the low-risk group was sensitive to immune checkpoint inhibitors, while the high-risk group exhibited sensitivity to mTOR inhibitors and polo-like kinase (PLK) inhibitors 77. High expression of HMGA1 is typically associated with adverse prognoses of cancer, and this study further revealed that ESCC cells with high HMGA1 expression were more sensitive to mTOR inhibitors, which indirectly supports the predictive findings reported by Jang et al 77.

Upregulation of HMGA1, while enhancing the self-renewal and invasive capabilities of the tumor, also sensitizes the tumor to specific drugs such as rapamycin. This implies that oncogenes might function as a double-edged sword for tumors, whereby they not only promote the proliferation and invasion of tumors but also render the tumors more therapeutic vulnerabilities. These results highlight the potential significance of HMGA1 as an indicator for targeted therapies and reinforce the importance of considering personalized treatment strategies based on individual risk profiles in EC patients.

Despite the remarkable responsiveness of specific patients to targeted therapies, the advancement of precision medicine remains a pending challenge owing to the shortage of indicator for patient selection and limited understanding of how to integrate targeted treatments with conventional therapies. However, as the molecular landscape of tumor initiation and progression becomes more comprehensive and with the advancements in liquid biopsy techniques, personalized precision treatments are gradually becoming feasible. At this juncture, by uncovering a novel molecular mechanism underlying ESCC's resistance to rapamycin, we undeniably contribute a missing puzzle piece to the map of resistance mechanisms and mark a significant step forward in achieving individualized treatment strategies.

Although FKBP12 serves as the intracellular receptor for rapamycin, there are limited researches into the impact of its expression levels on the therapeutic efficacy of rapamycin, especially within tumors. It wasn't until recently that Zhang et al. demonstrated a crucial role of FKBP12 expression in determining the sensitivity of glioblastomas to rapamycin treatment 78. However, factors regulating FKBP12 in tumor cells have remained poorly studied. In the current study, we delved into the upstream regulators of FKBP12 in ESCC and thoroughly explore the mechanisms through which HMGA1 upregulates FKBP12 expression and hence affects cellular sensitivity to mTOR inhibition.

Given that FKBP12 facilitates the intracellular accumulation of rapamycin, elevated expression of FKBP12 induced by HMGA1 in tumor cells offers the possibility of enhancing drug accumulation within the tumor. By coupling other therapeutic agents with the FKBP12-binding domain of rapamycin, increased drug accumulation within the tumor can be achieved. This strategy not only amplifies the therapeutic efficacy but also diminishes off-target toxicity, thereby addressing issues related to non-tissue-specific adverse effects. Further understanding of the interplay between FKBP12 and these drugs may lead to improved therapeutic strategies for a range of conditions and diseases. Drug resistance is a critical hurdle for an effective cancer therapy. To advance the progress of precision medicine, a more comprehensive evaluation of drug resistance in ESCC is necessary. CRISPR screens can help identify key genes involved in drug resistance. Sensitization of ESCCs to rapamycin by activation of the HMGA1-FKBP12 axis provides an efficient solution and proof-of-concept for overcoming drug resistance in ESCCs.

Through RNA-seq, we are able to unveil a portion of the regulatory network involving HMGA1. Among the DEGs, there is a significant enrichment in signaling pathways such as the PI3K-Akt signaling pathway, Hedgehog signaling pathway, ECM-receptor interaction, and others. However, this paper focuses on how HMGA1 regulates the sensitivity of ESCC cells to mTOR inhibitors. As a result, a comprehensive analysis of HMGA1's role in ESCC cells was not undertaken, and its full significance in this context remains to be explored.

## Supplementary Material

Supplementary figures.

## Abbreviations

ESCC: Esophageal squamous cell carcinoma
EC: Esophageal cancer
EAC: Esophageal adenocarcinoma
FRB: FKBP-rapamycin-binding
NIH: National Institutes of Health
ATCC: American type culture collection
SncRNA: small non-coding RNAs
ECL: Electrogenerated chemiluminescence
RIN: RNA integrity number
CHIP: Chromatin immunoprecipitation
EBS: ETS1 binding site
