# Supplementary material for: HMGA1 sensitizes esophageal squamous cell carcinoma to mTOR inhibitors through the ETS1-FKBP12 axis
Source: Int J Biol Sci. 2024 Apr 22;20(7):2640–57. doi: 10.7150/ijbs.95595 (PMC11077367; doi:10.7150/ijbs.95595)
Supplement: Supplementary file 1 — Supplementary figures. [file ijbsv20p2640s1.pdf]

Supplementary Figure

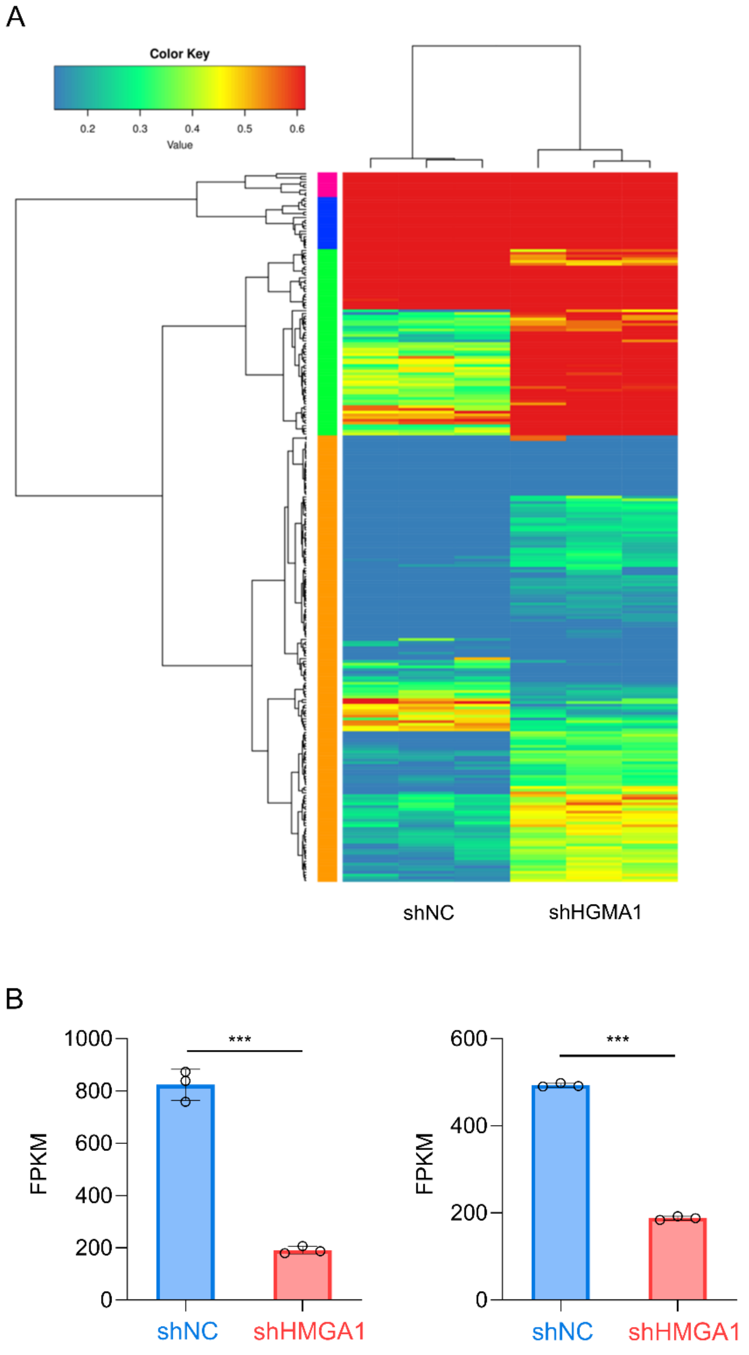

Supplementary Figure 1

Fig 1. Analysis of RNA-seq results after silencing HMGA1 in KYSE30 cells

A. Unsupervised hierarchical clustering separated cells with HMGA1 expression (controls) from those with HMGA1 silencing.

B. Fragments Per Kilobase of exon model per Million mapped fragments (FPKM) of

HMGA1 and *FKBP1A* by RNA-seq.

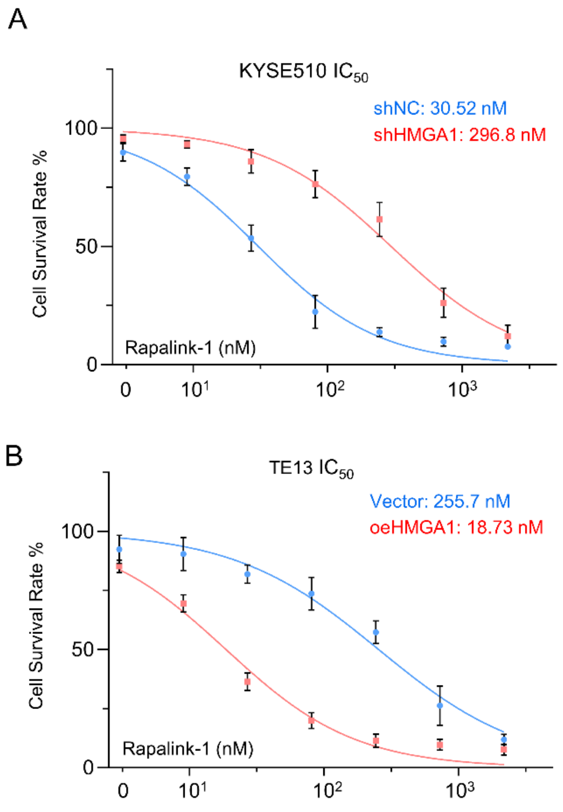

Supplementary Figure 2

Fig 2. IC50 of ESCC cells silenced or overexpressing HMGA1 in response to rapalink-1 treatment.

(B) KYSE510 cells with or without HMGA1 knockdown were treated with rapamycin in a concentration gradient (starting at 8 nM and increasing fivefold each time). Cell viability was measured using the CCK8 assay. **(D)** TE13 cells with or without HMGA1 overexpression were treated with rapamycin in a concentration gradient (starting at 8 nM and increasing fivefold each time). Cell viability was measured using the CCK8 assay.

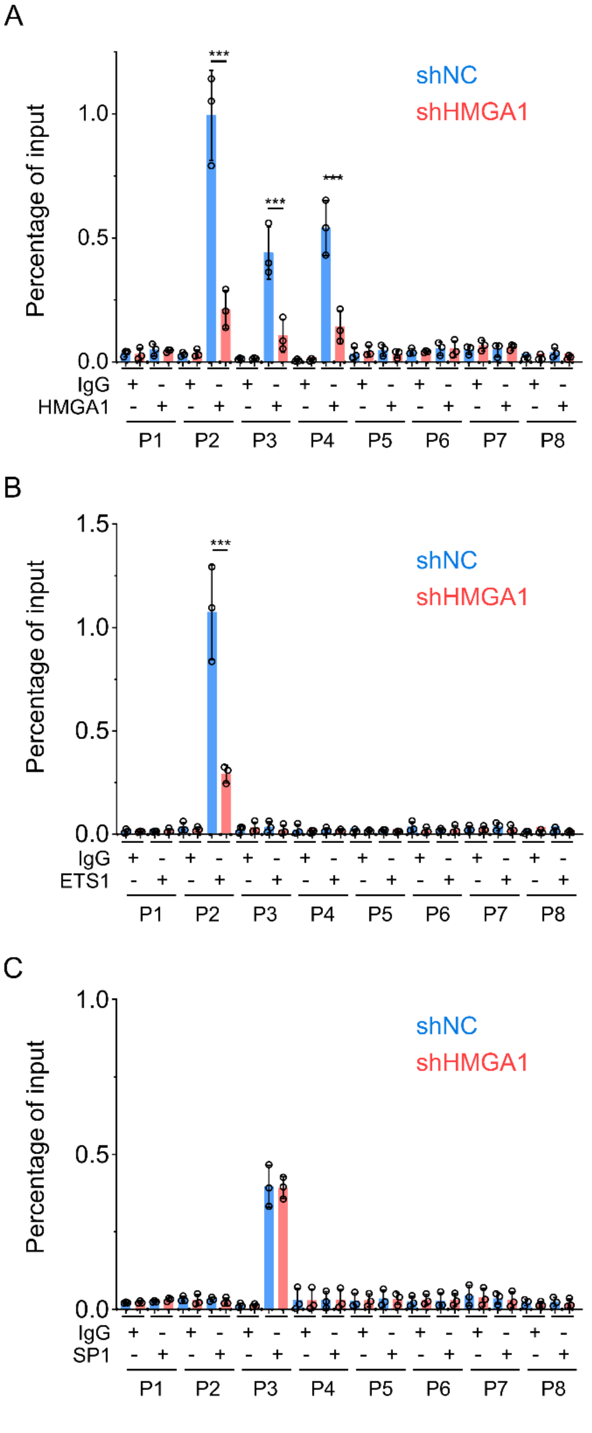

Supplementary Figure 2

Fig 2. Chromatin immunoprecipitation assay to analyze the binding of HMGA1/ETS1/SP1 in the FKBP12 promoter region

A. ChIP PCR was performed to detect the binding of HMGA1 to the FKBP1A promoter region in KYSE30 cells with or without HMGA1 silencing. HMGA1-chip antibody and

non-specific control IgG were used in the ChIP assay to assess HMGA1 binding to the target region. Results are presented as the percentage recovered from the total input DNA (% input) performed in triplicate in 3 independent experiments.

B. ChIP PCR was performed to detect the binding of HMGA1 to the FKBP1A promoter region in KYSE30 cells with or without HMGA1 silencing. ETS1-chip antibody and non-specific control IgG were used in the ChIP assay to assess HMGA1 binding to the target region. Results are presented as the percentage recovered from the total input DNA (% input) performed in triplicate in 3 independent experiments.

C. ChIP PCR was performed to detect the binding of HMGA1 to the FKBP1A promoter region in KYSE30 cells with or without HMGA1 silencing. SP1-chip antibody and non-specific control IgG were used in the ChIP assay to assess HMGA1 binding to the target region. Results are presented as the percentage recovered from the total input DNA (% input) performed in triplicate in 3 independent experiments.
